# Health insurance status and its determinants among patients with type 2 diabetes mellitus in a tertiary teaching hospital in Malaysia

**DOI:** 10.1371/journal.pone.0267897

**Published:** 2022-05-05

**Authors:** Essam Ali Al-Sanaani, Aniza Ismail, Mohd Rizal Abdul Manaf, Leny Suzana Suddin, Norlaila Mustafa, Norlela Sukor, Alabed Ali A. Alabed, Ahmed Abdelmajed Alkhodary, Syed Mohamed Aljunid

**Affiliations:** 1 Faculty of Medicine, Department of Community Health, Universiti Kebangsaan Malaysia, Kuala Lumpur, Malaysia; 2 Ministry of Public Health and Population, Sana’a, Yemen; 3 Faculty of Medicine, Department of Community Health, Universiti Teknologi MARA, Shah Alam, Selangor, Malaysia; 4 Faculty of Medicine, Department of Medicine, Universiti Kebangsaan Malaysia, Kuala Lumpur, Malaysia; 5 Faculty of Medicine, Department of Community Medicine, Lincoln University College, Kota Bharu, Malaysia; 6 Faculty of Medicine, International Centre for Casemix and Clinical Coding, National University of Malaysia, Kuala Lumpur, Malaysia; 7 Faculty of Public Health, Department of Health Policy and Management, Kuwait University, Kuwait, Kuwait; Management and Science University, MALAYSIA

## Abstract

**Introduction:**

Even in a country with a tax-based healthcare financing system, health insurance can play an important role, especially in the management of chronic diseases with high disease and economic burden such as Type 2 Diabetes Mellitus (T2DM). The insurance coverage among T2DM patients in Malaysia is currently unclear. The aim of this study was to determine the insurance status of T2DM patients in public and private healthcare facilities in Malaysia, and the association between this status and patients’ sociodemographic and economic factors.

**Methods:**

A cross-sectional study among T2DM patients seeking inpatient or outpatient treatment at a public tertiary hospital (Hospital Canselor Tuanku Muhriz) and a private tertiary hospital (Universiti Kebangsaan Malaysia Specialist Centre) in Kuala Lumpur between August 2019 and March 2020. Patients were identified via convenience sampling using a self-administered questionnaire. Data collection focused on identifying insurance status as the dependent factor while the independent factors were the patients’ sociodemographic characteristics and economic factors.

**Results:**

Of 400 T2DM patients, 313 responded (response rate, 78.3%) and 76.0% were uninsured. About 69.6% of the respondents had low monthly incomes of <RM5000. Two-thirds of participants (59.1%) spent RM100–500 for outpatient visits whilst 58.5% spent <RM100 on medicines per month (RM1 = USD0.244). Patients who visited a private facility had five times more likely to have insurance than patients who visited a public facility. Participants aged 18–49 years with higher education levels were 4.8 times more likely to be insured than participants aged ≥50 years with low education levels (2 times).

**Conclusions:**

The majority of T2DM patients were uninsured. The main factors determining health insurance status were public facilities, age of ≥ 50 years, low education level, unemployment, and monthly expenditure on medicines.

## Introduction

In Malaysia, the healthcare system has a two-tier structure. The first tier is a heavily funded public healthcare system that requires minimal out-of-pocket (OOP) payments, while the second tier is private healthcare services that are funded by patients through OOP expenditure, private health insurance, employer-funded health insurance or other employer-funded healthcare schemes [1,2].

As the Malaysian health system is mainly tax-based, people who do not have private insurance can still access healthcare in government facilities that charge with minimal fees [3,4]. However, due to the high workload at these facilities, it can be difficult for patients to receive treatment, e.g. long waiting times for consultation or surgery [5]. These shortcomings in the public healthcare system can have a very detrimental effect on the health of people with chronic diseases such as diabetes mellitus, heart disease or cancer [6,7]. It is possible that delays in treatment can lead to poorer health of patients [8]. It is therefore not surprising that patients with chronic diseases in Malaysia purchase private insurance schemes for the treatment of their illnesses [9].

Although the Malaysian health system is mainly tax-based, fees in public and private hospitals are generally higher for foreigners than those for locals. Foreigners seeking treatment at health facilities may incur high costs [10,11]. To financially protect migrant workers from the costs of inpatient treatment or surgery at Ministry of Health hospitals, the government has made membership in a health insurance scheme for foreign workers, the SPIKPA (Foreign Worker Hospitalisation and Insurance Scheme), mandatory since 2011 [12,13].

Therefore, health insurance is generally one of several healthcare financing mechanisms that may be able to prevent health-related financial disasters among those who contribute to the schemes [2]. The role of health insurance is important even in a country that has a tax-based system of healthcare financing.

### Diabetes and health insurance

Diabetes mellitus (DM) is a metabolic disease that is considered one of the most common chronic diseases worldwide [14–17]. The World Health Organisation (WHO) has classified DM as a non-communicable epidemic disease [18]. In 2015, there were 415 million diabetics aged 20–79 years worldwide, and this number is expected to increase to 642 million by 2040 [15]. Diabetes is expected to account for more than 16% of global healthcare expenditure in middle-income countries by 2040 [15].

T2DM is currently considered a global health and financial concern [19–21]. T2DM is associated with several morbidities: Cardiovascular disease is a major cause of mortality in about one-third (32.2%) of T2DM patients [22]. The disease is associated with an increased risk of hospitalisation [23].

T2DM is a costly disease to manage because of the high rate of diabetes-related complications. These complications of T2DM affect patients’ quality of life [24,25] and increase the economic burden on patients [25–27]. Several cost-related issues have been identified in the diabetes management literature, including medication costs, physician consultations, and laboratory investigations [28].

T2DM patients who have to pay OOP for health care services and medicine themselves may have high health expenditures. This contributes to financial problems that can push them into poverty [29]. Poverty, in turn, may lead diabetics to spend less money on other basic needs and forgo healthcare that would enable them to survive their illnesses [30,31].

The T2DM and its complications also result in reduced individual productivity and income due to early retirement, which increases the financial burden on both the individual and the government through the loss of labour productivity and increased expenditure on disease-related healthcare [32].

In Malaysia, T2DM is one of the most prevalent chronic diseases [33]. In the last decade, it has even been classified as an epidemic as the prevalence of T2DM has increased significantly in the country [34,35]. According to the 2019 National Health and Morbidity Survey (NHMS) report, the prevalence of DM in Malaysia has increased from 11.2% in 2011 to 13.4% in 2015 and 18.3% in 2019 [36], meaning that almost one in five Malaysian adults has diabetes. By 2025, seven million Malaysian adults are likely to have diabetes—an alarming trend that will lead to a diabetes prevalence of 31.3% among adults aged ≥18 years [37]. This has serious implications for the healthcare and social system and the national economy. Therefore, Malaysian health policymakers consider T2DM as a major public health concern [33].

In Malaysia, the government provides highly subsidised healthcare services to Malaysian patients in public hospitals. For example, Malaysian patients at Hospital Canselor Tuanku Muhriz (HCTM) pay only Malaysian Ringgit (RM)30 per visit for outpatient services and RM50 per day for inpatient services [38]. Although these treatment costs are considered low, some patients prefer to either pay themselves (OOP) or purchase insurance due to the aforementioned long waiting times and overcrowding at public healthcare facilities. In addition, there is a perception that private facilities provide high quality services.

Many studies have been conducted on the characteristics of people who purchase health insurance policies. Local studies have shown that health insurance status is positively associated with occupation. For example, people with higher incomes were willing to pay for premium health insurance [39,40]. In contrast, people with lower incomes found it difficult to pay for premium health insurance [40,41]. Mustafa et al. pointed out that this affected the demand for health insurance by 37.0% [42]. In additions, individuals with higher household incomes were more likely to have private insurances, which increases their awareness of the importance of insurance coverage as they protect their dependents against financial health disasters [43–45], compared to individuals with lower incomes [45,46].

Several studies among T2DM have shown that sociodemographic factors (age, gender, marital status, ethnicity, and education) and socioeconomic factors (household income and occupation) are associated with insurance status [39,41,42,47,48].

Since the main objective of policymakers in healthcare in any country is to reduce OOP expenditure on healthcare in general and chronic diseases such as DM in particular so that patients can access healthcare services without economic hardship [31], there is a need for a study that captures the characteristics of patients who purchase health insurance in Malaysia. This knowledge is important for the various stakeholders in the country’s healthcare system as it can help policymakers to review and design appropriate mechanisms to finance healthcare for people with chronic diseases. However, to date, there is a lack of findings on health insurance status among T2DM patients in Malaysia. Therefore, the aim of this study was to determine the health insurance status and its association with the sociodemographic and economic factors of T2DM patients in Malaysia.

## Materials and methods

### Data source

To ensure representativeness of diabetes patients in terms of those seeking treatment at public and private healthcare facilities, this study was conducted at Hospital Canselor Tuanku Muhriz (HCTM), a public tertiary hospital under the Ministry of Higher Education, Malaysia, and its private arm, the Universiti Kebangsaan Malaysia Specialist Centre (UKMSC). Both facilities are located in Kuala Lumpur. At these facilities, participants for this study were recruited from both the outpatient (endocrine clinics) and inpatient (wards) facilities.

### Sample size and sampling

The sample size was calculated using Pocock’s sample size [49]. The minimum sample size, calculated for a 95% confidence level (95% CI), was 400 patients. The sample population included all T2DM patients who had consented to participate in the study, patients who had visited the endocrine clinic, and patients who had been hospitalised at the two hospitals during the data collection period.

The inclusion criteria were T2DM diagnosis, under treatment, or follow-up at HCTM and UKMSC. All participants were Malaysians, aged ≥18 years, and able to understand and communicate in English or Malay language. Exclusion criteria were refusal to participate in the study and disability (visual, speech, or hearing disability). Data were collected using a convenience sampling approach.

### Measurements and questionnaires

The study tool was a validated questionnaire adapted from a previous study [50]. The questionnaire used in this current study was in English and Malay and was translated from English to Malay and vice versa, underpinned by face validity and content validity. Content validity was determined by two local public health and health economics specialists, while face validity was determined by diabetic patients who attended another health center. The questionnaire consists of two sections. The first section of the questionnaire collected data on sociodemographic characteristics such as age, gender, ethnicity, marital status, and education level. The second section collected data on economic factors, namely occupation, household income, household expenditure and health insurance status (i.e., insured and uninsured), healthcare expenditure per visit, and monthly expenditure on medicines.

In the data collection tool, the patient’s health insurance status was the dependent factor, while the independent factors were the patient’s sociodemographic characteristics and economic factors.

### Definition of variables

Health insurance status in this study can be either “insured” or “uninsured”. Health insurance status refers to the current status of respondents, i.e. whether they have active private health insurance (insured) or whether they do not have active private health insurance and instead paid their medical bills through OOP or government subsidies (uninsured).

Patients were defined as fully insured if they did not make any payments for medical services, as insurance companies covered all their medical expenses. Patients were defined as partially insured if they had partial health insurance, where the insurance companies only covered part of the patient’s medical costs and patients had to share the cost of their medical treatments with the health insurance company through co-payment. The currency used in the study was the Malaysian Ringgit, with an exchange rate of RM1 = USD 0.244 in 2020.

### Data collection

Data collection was conducted by the researchers and trained research assistants from August 2019 until March 2020. Each patient was fully informed about the aim of the study and its procedures before answering the questions. Written informed consent was obtained from each patient before the questionnaire was administered to the consenting patient. Data collection took place on days when the endocrine clinic was in operation and on weekdays in the hospital wards.

### Ethical approval and consent to participate

The study was approved by the UKM Medical Research Ethics Committee (reference: UKMPP1/111/8/JEP.2019.186), and by the HCTM and UKMSC management. Written informed consent was obtained from the participants and the consent form was approved by the ethics committees. All study-related methods were performed in accordance with the applicable guidelines and regulations in Malaysia.

### Statistical analysis

Data were analysed using the Statistical Package for the Social Sciences (SPSS, version 25). The sociodemographic and economic variables were identified using descriptive statistics and frequency tables. Pearson’s Chi-square test was performed for the bivariate analysis and multiple logistic regressions were performed for the multivariable analysis. The significance level of p < 0.05 was established for the entire analysis.

## Results

### Characteristics of the Participants’

A total 313 out of 400 T2DM patients responded to the questionnaire, representing a response rate of 78.3%. Of these, 235 respondents were from HCTM while 78 were from UKMSC. Eighty-seven patients were classified as non-respondents as they belonged to the group of patients who had not completed the questionnaires and had not returned the questionnaires.

**Fig 1:**
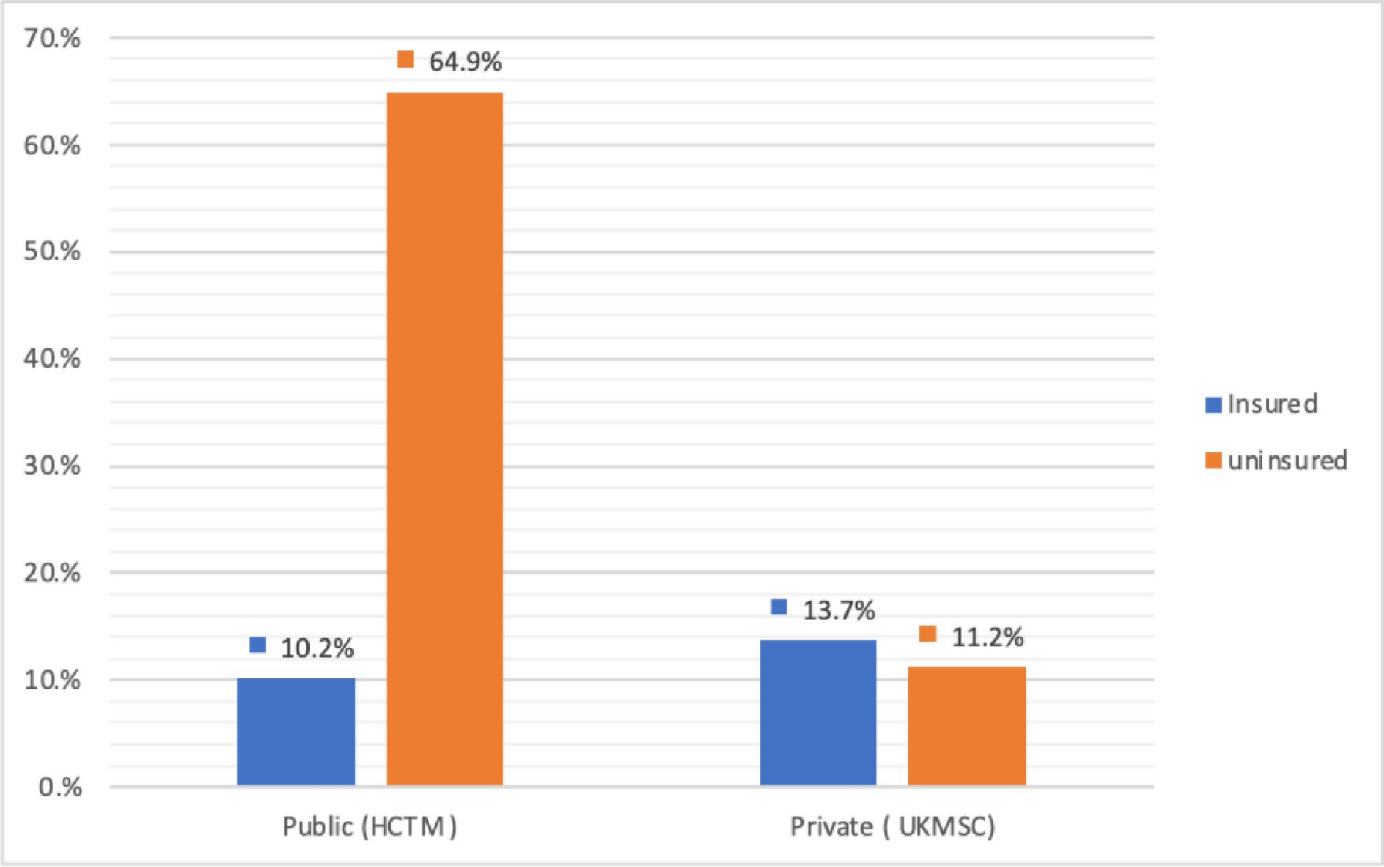
Insurance status of T2DM patients at HCTM and UKMSC.

A total of 76.0% of the patients (n = 238/313) were uninsured. Most of the patients treated at HCTM (n = 203/235, 86.4%) were uninsured. In contrast, about half of the UKMSC-treated patients (n = 43/78, 55.1%) were insured. Fig 1 illustrates the insurance status of the patients with HCTM and UKMSC. Of the total sample, 64.9%-HCTM and 11.2%-UKMSC patients were uninsured, while 10.2%-HCTM and 13.7%-UKMSC patients were insured.

Table 1 shows the distribution of respondents by sociodemographic characteristics. Most (n = 235/313; 75.1%) were treated for HCTM. About half (52.1%) of the respondents were from outpatient clinics. Up to 53.0% of the sample consisted of female patients. Most respondents were ≥50 years old (76.4% of the total sample) and most were married (89.1%). Malay patients accounted for 64.2% of the total sample, while Chinese and Indian patients accounted for 17.0% and 18.8%, respectively. Of the total sample, 64.5% of patients had a low level of education.

**Table 1:** Distribution of respondents by sociodemographic characteristics.

Variable	n (%)
**Service provider**	
HCTM	235 (75.1)
UKMSC	78 (24.9)
**Source of patient**	
Inpatient	150 (47.9)
Outpatient	163 (52.1)
**Gender**	
Male	147 (47.0)
Female	166 (53.0)
**Age Group**	
18-49years	74 (23.6)
50 years & above	239 (76.4)
**Marital Status**	
Married	279 (89.1)
Unmarried	34 (10.9)
**Ethnicity**	
Malay	201 (64.2)
Chinese	53 (17.0)
Indian	59 (18.8)
**Education Level**	
Low education	202 (64.5)
High education	111 (35.5)

Table 2 shows the distribution of respondents by socioeconomic characteristics. Most respondents were uninsured, accounting for 76.0% of the total sample. Data on the occupational status of respondents show that the highest percentage (33.5%) were pensioners. Respondents with a household income of <RM5000 accounted for 69.6% of the total sample. Up to 80.5% of the respondents had monthly family expenditure of <RM5000 and 59.1% of the respondents spent between RM100 and RM500 per visit. In addition, 58.5% of the respondents spent <RM100 per month on medicines.

**Table 2:** Distribution of respondents by socioeconomic characteristics.

Variable	n (%)
**Health Insurance Status**	
Insured	75 (24.0)
Uninsured	238 (76.0)
**Occupation**	
Private	68 (21.7)
Government	40 (12.8)
Unemployed	100 (32.0)
Pensioner	105 (33.5)
**Household Income**	
<RM5000	218 (69.6)
≥ RM5000	95 (30.4)
**Family Expenditure**	
< RM5000	252 (80.5)
≥ RM5000	61 (19.5)
**Treatment Spending/ Visit**	
< RM100	70 (22.4)
RM100—RM500	185 (59.1)
> RM 500	58 (18.5)
**Medicine Spending/ month**	
< RM100	183 (58.5)
RM100—RM500	119 (38.0)
> RM500	11(3.5)

#### Bivariate analysis results

A Chi-square test for independence was performed to assess the relationship between participants’ health insurance status and sociodemographic characteristics. The results are summarised in Table 3. In terms of study sites (HCTM versus UKMSC), the results showed that most HCTM-treated participants were uninsured (n = 203/235; 86.4%) compared with UKMSC-treated patients (n = 35/78; 44.9%). Chi-square test showed that health insurance status was significantly associated with study site [χ^*2*^ (1, N = 313) = 55.386, p < 0.001].

**Table 3:** Association between health insurance status and sociodemographic characteristics.

Variable	n	Health insurance status		P-value
		Insured, n (%)	Uninsured, n (%)	
**Service Provider**				
HCTM	235	32 (13.6)	203 (86.4)	<0.001*
UKMSC	78	43 (55.1)	35 (44.9)	
**Source of patients**				
Inpatient	150	21 (14.0)	129 (86.0)	<0.001*
Outpatient	163	54 (33.1)	109 (66.9)	
**Gender**				
Male	147	38 (25.9)	109 (74.1)	0.461
Female	166	37 (22.3)	129 (77.7)	
**Age Groups**				
18–49 years	74	37 (50.0)	37 (50.0)	<0.001*
50 & above	239	38 (15.9)	201 (84.1)	
**Marital status**				
Unmarried	34	10 (70.6)	24 (29.4)	0.276
Married	279	65 (23.3)	214 (76.7)	
**Ethnicity**				
Malay	201	59 (29.4)	142 (70.6)	0.006*
Chinese	53	10 (18.9)	43 (81.1)	
Indian	59	6 (10.2)	53 (89.8)	
**Educational level**				
^1^Low	202	29 (14.4)	173 (85.6)	<0.001*
^2^High	111	46 (41.4)	65 (58.6)	

*Significant P value<0.05

^1^Low = none, primary school, secondary school

^2^High = professional higher education or university; HCTM–public facility, UKMSC–private facility.

Similarly, in terms of patient source (outpatient versus inpatient facility), 66.9% (n = 109/163) of respondents treated in outpatient clinics were uninsured compared with 86% (n = 129/150) of respondents treated in inpatient wards. The chi-square test showed that health insurance status was significantly associated with patient source [χ^*2*^ (1, N = 313) = 15.688, p < 0.001].

Regarding age, the analysis showed that 84.1% (n = 201/239) of respondents aged ≥50 years were uninsured compared to only 50.0% (n = 37/74) of those aged 18–49 years. The chi-square test showed that health insurance status was significantly associated with age group [χ^*2*^ (1, N = 313) = 36.062, p < 0.001].

Ethnicity is also a significant factor associated with health insurance status. A total of 89.8% (n = 53/59) of Indian respondents were uninsured compared to 70.6% (n = 142/201) of Malays and 81.1% (n = 43/53) of Chines. Again, the chi-square test showed that health insurance status was significantly associated with ethnicity [χ ^*2*^ (2, N = 313) = 10.121, p = 0.006]. In addition, 85.6% (n = 173/202) of respondents with low education level were uninsured compared to 58.6% (n = 65/111) with high education level; health insurance status was significantly associated with education level [χ ^*2*^ (1, N = 313) = 28.843, p<0.001].

**Fig 2:**
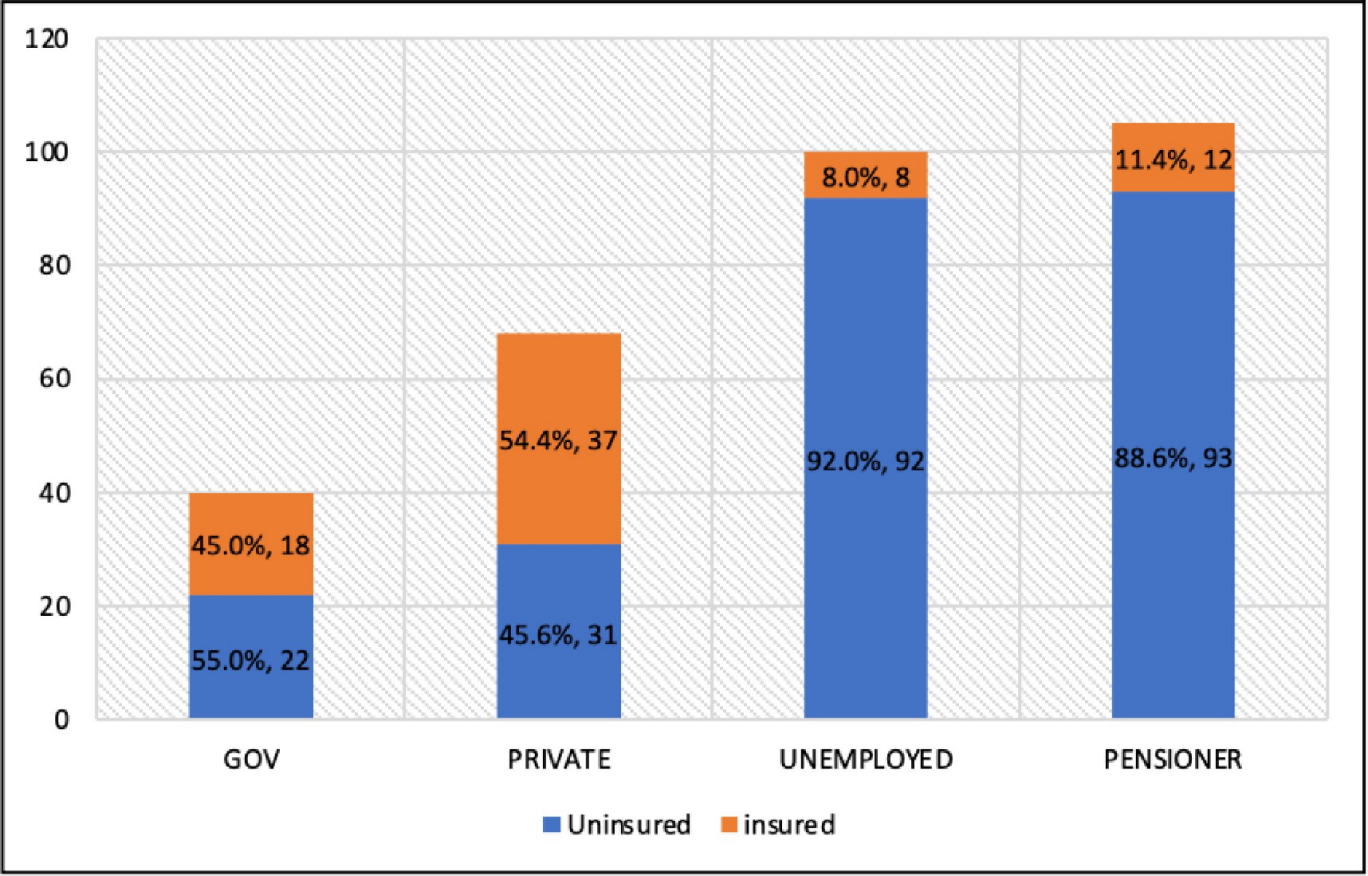
Distribution of participants’ occupation and association with health insurance status among T2DM patients at UKM teaching hospital.

Fig 2 shows the distribution of occupations among participants and the association between occupation and health insurance status. Pensioners accounted for the largest portion of respondents, representing 33.5% of the total sample. In addition, 88.6% of pensioners were not covered by health insurance. Participants who worked in the private sector had higher health insurance coverage, 54.4% of all respondents. The Chi-square test showed that health insurance status was significantly associated with occupation status [χ ^*2*^ (3, N = 313) = 67.357, p<0.001].

Table 4 explains the association between the respondents’ health insurance status with other economic factors. As many as 85.8% of respondents with a monthly household income of <RM5000 did not have health insurance, as did 82.5% of respondents with monthly family expenses of <RM5000. Chi-square test showed that health insurance status was significantly associated with household income and family expenditure [χ ^*2*^ (1, N = 313) = 37.4, p<0.001; χ ^*2*^ (1, N = 313) = 29.997, p<0.001, respectively]. Regarding monthly expenditure on medicines, 82.0% of the respondents who spent <RM100 per month on medicines were uninsured. Chi-square test showed that health insurance status was significantly associated with monthly expenditure on medicines [χ ^*2*^ (1, N = 313) = 14.974, p = 0.001].

**Table 4:** Association between health insurance status, income and health spending.

Variables	N	Health insurance status		P-value
		Insured, n (%)	Uninsured, n (%)	
**Household Income/Month**				
< RM 5000	218	31 (14.2)	187 (85.8)	<0.001*
≥ RM 5000	95	44 (46.3)	51 (53.7)	
**Family Expenditures/Month**				
< RM5000	252	44 (17.5)	208 (82.5)	<0.001*
≥ RM5000	61	31(50.8)	30 (49.2)	
**Treatment Spend Per Visit**				
Less than RM100	70	10 (14.3)	60 (85.7)	0.086
RM 100—RM500	185	51(27.6)	134 (72.4)	
Above RM500	58	14 (24.1)	44 (75.9)	
**Medicine Spending/Month**				
Less than RM100	183	33 (18.0)	150 (82.0)	<0.001*
RM100—RM500	119	35 (29.4)	84 (70.6)	
Above RM500	11	7 (63.6)	4 (36.4)	

*Significant P value < 0.05.

### Multivariate analysis

Table 5 shows that the probability of being insured was five times higher among UKMSC participants compared to HCTM participants [95% CI: 2.4–10.7]. Respondents aged 18–49 years were 4.8 times more likely to be insured compared to respondents aged ≥50 years [95%CI: 2.4–9.6]. Respondents with a high level of education were twice as likely to be insured as respondents with a low level of education [95% CI: 1.02–3.74].

**Table 5:** Multivariate analysis of health insurance status for sociodemographic and economic factors.

	Reference group	B	Adjusted OR	95%CI		P-value
				lower	upper	
**Service provider **						
UKMSC (Private facility)	HCTM (Public facility)	1.61	5.01	2.4	10.7	<0.001*
**Age groups **						
18–49 years	50 years &above	1.56	4.77	2.38	9.55	<0.001*
**Education level**						
High education	Low education	0.67	1.96	1.02	3.74	0.042*
**Occupational status**						<0.001*
Government	Private	- 0.311	0.733	0.319	1.685	0.464
Unemployed		-2.267	0.104	0.042	0.257	<0.001*
Pension		-1.805	0.165	0.073	0.373	<0.001*
**Medicine spending/month**						0.041*
RM100-RM500	< RM100	0.526	1.692	0.896	3.195	0.105
> RM500		1.714	5.552	1.221	25.235	0.026*

*Significant P value < 0.05.

Unemployed respondents and pensioners were 10.4% and 16.5% less likely to be insured than respondents who worked in the private sector [95% CI: 0.042–0.257; 95% CI: 0.073–0.373, respectively]. Respondents who spent more than RM500 per month on medicines were 5.6 times more likely to be insured than those who spent less than RM100 per month on medicines [95% CI: 1.22–25.2].

## Discussion

Our study shows that the majority of T2DM patients treated at a public facility (HCTM) were uninsured. This could be due to the Malaysian government’s efforts to minimise the financial burden of healthcare expenditure on citizens by subsidizing healthcare services in public facilities. This explains why most Malaysian citizens prefer to visit public healthcare facilities rather than private ones, as the Malaysian government bears their health expenditure with a minimal fee [51–55].

Our findings also show that there is a significant association between the insurance status, healthcare provider, source of patients, age group, ethnicity, and education level, which is consistent with the Balqis-Ali et al. [41]. In addition, it was found that the low insurance rate among Malaysians is due to the fact that the government heavily subsidises the cost of health care through universal health coverage, enrolment in health insurance is voluntary and most Malaysians are not willing to pay for their healthcare services [56]. These findings are consistent with those of previous studies in Malaysia, which found that 26.8% of Malaysians were privately insured [41,57].

Here, we found a significant association between health insurance status and age group, with the majority of participants with insurance coverage being in the younger age group [41]. It is worth noting that older people may need health insurance coverage to ensure easy access to medical care without financial burden [58,59]. According to Arsenijevic et al, (2016) and Chiew, (2017), older people are highly vulnerable to financial shocks as they often have low incomes. Our data show that older people very rarely purchase insurance. This could be due to the fact that low-income elderly people are not aware of the importance of health insurance [41,60,61]. Another reason for this result could be the highly subsidised public health services provided by the government [53,62]. It is worth noting that 77.8% of the elderly participants had a household income of less than RM5000 compared to 33.2% of the younger participants. This decreases the likelihood of having health insurance [41,61], while higher income leads to a better access to health insurance [39,46,63–65].

Our data are also consistent with previous studies that have shown that low-income people are less likely to purchase health insurance. For example, in 2015, Nelson and colleagues [66] found that uninsured people were more likely to be in the low-income group and were often poorer, unemployed and with low levels of education [67].

We also found out that there is a significant association between enrollment in health insurance and the level of education: Respondents with a high level of education had a higher enrolment rate in health insurance than respondents with a low level of education [43,68,69]. Moreover, our results are consistent with those of Mustafa et al, (2019), who reported that a higher level of education increases enrolment in the health insurance system [42]. It is well known that there is a positive association between health insurance enrolment patterns and awareness of the importance of health insurance in protecting against health-related financial disasters [70–74]. In addition, patients with high income and education levels prefer to seek health services in private facilities, which may be due to the fact that quality healthcare services are easier to obtain in private facilities than in public facilities [46]. Our findings are consistent with previous studies that have shown that patients with higher levels of education and income are more likely to be able to pay their health care expenses than others [41,46,75]. Greater awareness of the importance of protecting family members from health-related financial disasters leads to greater willingness to enroll in health insurance programmes [43,44,72,76].

Our study showed that there is a significant association between health insurance status and occupation [41,77]. Here, the pensioners and unemployed participants had a higher probability of being uninsured. However, participants who work in the private sector had a higher probability of having health insurance than participants in other occupations [41]. The pensioners are likely to be in the low-income category; this may be their barrier to paying health insurance premiums [78]. In addition, financial disasters may delay patients’ treatments and lead to diabetes-related complications [79] and diabetes-related hospitalisations, further burdening their financial status [8,80].

Our results show that 24% of the participants had health insurance overall. This can be interpreted to mean that these participants prefer to use private healthcare services, which are easier to obtain [41]. For example, private healthcare services are faster and have shorter waiting times in outpatient and routine surgeries than public facilities [75,81,82].

## Limitations of the study

To date, this is the first study on the health insurance status of T2DM patients in Malaysia. However, there are some limitations in this study: Firstly, the data only reflect the experiences of two sites (public and private departments) UKM Teaching Hospital. Therefore, our findings may not be generalisable to other hospitals at other sites. It is worth noting that UKM Teaching Hospital is considered one of the largest referral hospitals in Malaysia and treats a large number of diabetic patients [83]. Secondly, convenience sampling may not be the best way to comprehensively represent our findings; we opted for this approach after facing a low response rate from patients. Originally, all HCTM and UKMSC patients with T2DM were selected using a systematic random sampling approach. However, due to the low response rate and lack of patients willing to participate in this study, we changed the sampling method to convenience sampling. The sample of the present study sample was the largest that could be reached during the study period. Nevertheless, we believe that it accurately reflects the health insurance status of HCTM and UKMSC patients. In addition, we had a problem with patient recruitment as some of the HCTM and UKMSC patients declined to participate in the study. Therefore, we recommend future studies in other Malaysian healthcare facilities T2DM patients to extend our findings. It was expected that the data obtained through the survey would have recall bias. We attempted to overcome this problem by asking the respondents the questions in laymen’s terms and by giving them some hints so that they had enough time to think before answering the questions.

## Conclusions

Based on the discussion of this study, it can be concluded that most T2DM patients were uninsured. They were more likely to attend public facilities, were of Indian ethnicity, and belonged to the age group ≥50-year. They had lower levels of education, were mostly unemployed, had low household incomes and family expenses, and spent <RM100 per month on medicines. On the other hand, T2DM patients who had insurance were more likely to be treated in a private facility. They were of Malay ethnicity and belonged to the 18–49 age group. They generally had higher levels of education, worked in the private sector, had high household incomes and family expenses, and spent >RM500 per month on medicines. If the services in the private facility are indeed better than those in the public facility, then according to the findings of this study, there is an inequity in the treatment of T2DM as only those who can afford the private services (including the insured) have access to those services. It is therefore recommended that further studies be carried out to determine perceptions of service and treatment quality and to inform future strategies for allocating financial resources in healthcare, including raising awareness among the population about the benefits of various health insurance schemes. Finally, our findings should also be relevant for policymakers and provide useful information for the future improvement of the healthcare system.

## Abbreviations

UKM: Universiti Kebangsaan Malaysia (National University of Malaysia)
HCTM: Hospital Canselor Tuanku Muhriz
DM: diabetes mellitus
T2DM: Type2 Diabetes Mellitus
OOP: Out-Of-Pocket
OECD: Organisation for Economic Co-operation and Development
RM: Malaysian ringgit
NHMS: National Health and Morbidity Survey
